# Implications of nutritional stress as nestling or fledgling on subsequent attractiveness and fecundity in zebra finches (*Taeniopygia guttata*)

**DOI:** 10.7717/peerj.3628

**Published:** 2017-08-21

**Authors:** Mariam Honarmand, E. Tobias Krause, Marc Naguib

**Affiliations:** 1 Department of Animal Behaviour, Bielefeld University, Bielefeld, Germany; 2 Institute of Animal Welfare and Animal Husbandry, Friedrich-Loeffler-Institut, Celle, Germany; 3 Behavioural Ecology Group, Wageningen University & Research, Wageningen, The Netherlands

**Keywords:** Mate choice, Zebra finch, Early developmental stress, Female choice, Male choice

## Abstract

The conditions an organism experiences during early development can have profound and long lasting effects on its subsequent behavior, attractiveness, and life history decisions. Most previous studies have exposed individuals to different conditions throughout development until nutritional independence. Yet under natural conditions, individuals may experience limitations for much shorter periods due to transient environmental fluctuations. Here, we used zebra finches (*Taeniopygia guttata*) in captivity to determine if conditions experienced during distinctly different early developmental phases contribute differently to male and female attractiveness and subsequent reproduction. We conducted a breeding experiment in which offspring were exposed to food regimes with (a) low quality food provided only during the nestling period, (b) low quality food provided only during the fledgling period, or (c) high quality food throughout early development. We show that despite short-term effects on biometry and physiology, there were no effects on either male or female attractiveness, as tested in two-way mate choice free-flight aviary experiments. In a subsequent breeding experiment, the offspring from the initial experiment were allowed to breed themselves. The next generation offspring from mothers raised under lower quality nutrition as either nestling or fledging were lighter at hatching compared to offspring from mothers raised under higher quality nutrition whereas paternal early nutrition had no such effects. The lack of early developmental limitations on attractiveness suggests that attractiveness traits were not affected or that birds compensated for any such effects. Furthermore, maternal trans-generational effects of dietary restrictions emphasize the importance of role of limited periods of early developmental stress in the expression of environmentally determined fitness components.

## Introduction

The conditions an animal experiences in early life can have profound and long lasting effects on its performance and fitness (Hales & Barker, 2001; Metcalfe & Monaghan, 2001; Lummaa & Clutton-Brock, 2002; Monaghan, 2008; Sachser, Hennessy & Kaiser, 2011; Crino & Breuner, 2015; Taborsky, 2017). Such long-term consequences of early developmental stress have been well studied in many taxa (Mousseau & Fox, 1998) and can be negative effects of poor developmental conditions or may be specifically evolved adaptations to harsh environments with limited resources (Hales & Barker, 2001; Wells, 2003; Wells, 2007; Hanson & Gluckman, 2014). Effects of early developmental stress also have been shown to project into the following generation (Lummaa & Clutton-Brock, 2002; Naguib, Nemitz & Gil, 2006; Krause & Naguib, 2014) and even to have horizontal social effects, affecting the survival of the partner (Monaghan et al., 2012).

Birds have been a key model for experimentally studying effects of early environmental constraints on physiology, behavior, and life history (Price, 1998; Lindström, 1999; Chaby, 2016), as here offspring develop without a direct physiological link to the mother, as in mammals. Altricial birds develop within about one month from a dependent nestling to a fully-grown and nutritionally independent juvenile. During this period of rapid growth, individuals are especially vulnerable to resource limitations such as food restrictions. These have been shown to have striking effects on various physiological, behavioural, and life history traits including trans-generational effects affecting subsequent offspring traits and reproduction (Boag, 1987; Birkhead, Fletcher & Pellat, 1999; Kitaysky et al., 1999; Buchanan, 2000; Nowicki et al., 2000; Kitaysky et al., 2001; Naguib & Gil, 2005; Spencer et al., 2005; Naguib, Nemitz & Gil, 2006; Brust et al., 2014; Krause & Naguib, 2014; Farrell, Kriengwatana & MacDougall-Shackleton, 2015; Krause, Krüger & Schielzeth, 2017). While most of these studies show negative effects of early developmental stress, also positive effects on fitness (Crino et al., 2014a) and positive effects on exploration and learning have been reported (Kitaysky et al., 1999; Krause et al., 2009; Crino et al., 2014b; Van Oers et al., 2015).

Yet experiencing food limitations during the whole period of nutritional dependence from parents is certainly a long time for a developing organism. Shorter periods of food limitations such as during bad weather or due to non-optimal timing of reproduction with respect to temporal variation in food availability may even be more common (Visser, Holleman & Gienapp, 2006; Reed, Jenouvrier & Visser, 2013). Offspring that experience short term food limitations, such as only during the nestling or fledgling period, might be better able to compensate for such shorter periods of developmental stress. Several studies showed that developmental stress during just the nestling or the fledging stage leads to short-term biometric effects and an increase in corticosterone (Criscuolo et al., 2008; Honarmand, Goymann & Naguib, 2010; Kriengwatana et al., 2014). Yet even when phenotypic effects of developmental stress appear to be transient, the potential costs in compensating for poor developmental conditions then may become apparent at later life-history stages (Metcalfe & Monaghan, 2001; Fisher, Nager & Monaghan, 2006; Criscuolo et al., 2008; Krause & Naguib, 2011; Hector & Nakagawa, 2012).

Studies exposing offspring to developmental stress during the whole period of nutritional dependence from the parents showed, for instance, that males then produce less developed sexually selected plumage ornaments and show limits in song learning or song production (De Kogel & Prijs, 1996; Nowicki, Peters & Podos, 1998; Spencer et al., 2003; Naguib & Nemitz, 2007; Hill, 2014; Peters, Searcy & Nowicki, 2014). Accordingly, such individuals might be less attractive as mate (De Kogel & Prijs, 1996) even though not all studies found such effects (Naguib, Heim & Gil, 2008). Likewise, females may be affected in their song preference (Riebel, 2009) and have been shown to have a lower preference strength for preferred songs or even prefer songs of males with similar developmental background and thus potentially higher behavioural compatibility (Kitaysky et al., 1999; Riebel, Naguib & Gil, 2009; Holveck & Riebel, 2010; Holveck, Geberzahn & Riebel, 2011; Honarmand, Riebel & Naguib, 2015). Specifically in monogamous species with biparental care, like many songbirds, males should also be choosy, as they need to find a compatible mate (Griffith, Owens & Burke, 1999; Amundsen, 2000; Jones, Monaghan & Nager, 2001). Yet few experiments have been conducted on male choice (Holveck, Geberzahn & Riebel, 2011; Zandberg et al., in press) and specifically it remains to be shown if also males discriminate between females with different early developmental backgrounds.

In order to determine if shorter term nutritional limitations, i.e., during either the nestling or fledgling period, affect attractiveness and fecundity of both sexes, we raised zebra finches (*Taeniopygia guttata*) under one of three experimental treatments during their first month after hatching (Honarmand, Goymann & Naguib, 2010). These shorter periods of nutritional restrictions were chosen to simulate naturally occurring shorter periods of lower food availability such as during of bad weather or when breeding is very early or very late relative to food availability in periods of breeding. Subjects received either (a) a low quality diet as nestlings followed by a high quality diet as fledglings, (b) a high quality diet as nestlings followed by a low quality diet as fledglings, or (c) a high quality diet throughout the whole first month. Effects of the nutritional treatment on offspring biometry, physiology, and foraging behaviour have been shown previously for these subjects from this experiment (Krause et al., 2009; Honarmand, Goymann & Naguib, 2010). For example, we showed that birds with nutritional restrictions during either period had a slower growth rate during the period of food restriction but then compensated in body mass until sexual maturity (Honarmand, Goymann & Naguib, 2010). These birds also showed elevated corticosterone levels during the period of food restriction (Honarmand, Goymann & Naguib, 2010). The effects of nutritional restrictions during the nestling period then resurfaced in adult life, after being exposed as adults to short term food deprivation, in form of a higher body mass loss and more explorative behaviour (Krause et al., 2009). Here we tested adult male and female attractiveness in an aviary mate choice experiment by giving non-experimental opposite sex individuals the opportunity to choose between two subjects raised under the different treatments mentioned above. Zebra finches form lifelong pairs and both parents invest equally in raising young (Griffith & Buchanan, 2010), so that we expect both partners to be choosy. In a subsequent breeding experiment, subjects were then paired to non-experimental opposite sex mates to test their reproductive success. We compared male and female reproductive success separately. Assuming that nutritional restrictions during the earlier developmental periods when offspring have steep growth curves are more severe, we predicted that both males and females would be less attractive when having experienced poorer nutritional conditions earlier in life, and that offspring from the earlier poorer nutritional treatments would also take longer to reproduce and produce smaller offspring when given the opportunity to breed as adults.

## Methods

The main breeding and mate choice experiments were conducted in May and June 2006 on non-domesticated wild-type zebra finches (Hoffman et al., 2014) from Australian origin at the University of Bielefeld, Germany (ca. F7 generation after birds were important from Australia). The second breeding experiment was conducted in 2007. For the main breeding experiment, subjects had been raised by their genetic parents in their natal brood but differed in diet quality provided to their parents during offspring rearing as described for this experiment in Honarmand, Goymann & Naguib (2010). Breeding of pairs took place in cages (83 × 30 × 40 cm) with attached wooden nest boxes (15 × 15 × 15 cm) and coconut fibers at the ground as nesting material. We allowed 86 pairs of unrelated birds to breed, out of which 36 pairs produced 149 hatchlings (day 0); 35 pairs then raised 108 fledglings (day 17), out of which 33 pairs raised 96 offspring until independence (day 35). Such a proportion of successful broods is not uncommon in captive zebra finches (Griffith et al., 2016) but here was not explained by breeding experience (successful broods (day 35): 16 pairs with both partners having had breeding experience; four pairs with one partner having had breeding experience, 13 pairs with both partners being first time breeders; unsuccessful pairs (day 35): 20 pairs with both partners having had breeding experience, seven pairs with one partner having had breeding experience, 26 pairs with both partners being first time breeders). One treatment group received lower quality food when offspring were nestlings, from day three until day 17, and higher quality food when they were fledglings, from day 17 until day 35 (Group LH (low-high)). The other treatment received higher quality food when offspring were nestlings, from day three until day 17, and lower quality food from day 17 until day 35 (Group HL (high-low)). As a positive reference a third group was provided with high quality food throughout the whole first month of the offspring (HH, high-high). All treatments started at day three of the oldest chick in a brood; until then high quality diet was provided to all birds. We did not include a group with poorer food throughout the first month, due to expected high offspring mortality under these conditions. Lower quality food consisted of seed mix and water *ad libitum*. Groups in the higher quality treatment additionally received millet, germinated seeds and commercial egg food (CéDé, Evergern, Belgium) daily. Furthermore, salads, greens, fruits and vegetable were provided three times weekly to the high quality group. Twice per week, water was enriched with vitamins in both groups. Details of protein, fat and fiber content are given in Honarmand, Goymann & Naguib (2010). By the time of nutritional independence (day 35 of the youngest of a brood) until day 65, offspring were assigned to mixed sex song tutor groups (in total 10 different tutor groups) to learn their song from the adult male in their tutor group. Each of these song tutor groups consisted of a different male song tutor and an adult female, which were both unrelated to the tutees, and six to 11 tutees, which originated from different nutritional groups. After day 35, all subjects received a diet of intermediate quality (daily *ad libitum* provisioning with dried and germinated seeds and fresh water (plus vitamins) and twice a week also egg food) and stayed together until they were at least six months old. At day 65 the tutors and the females were removed from the different tutor groups.

**Figure 1:**
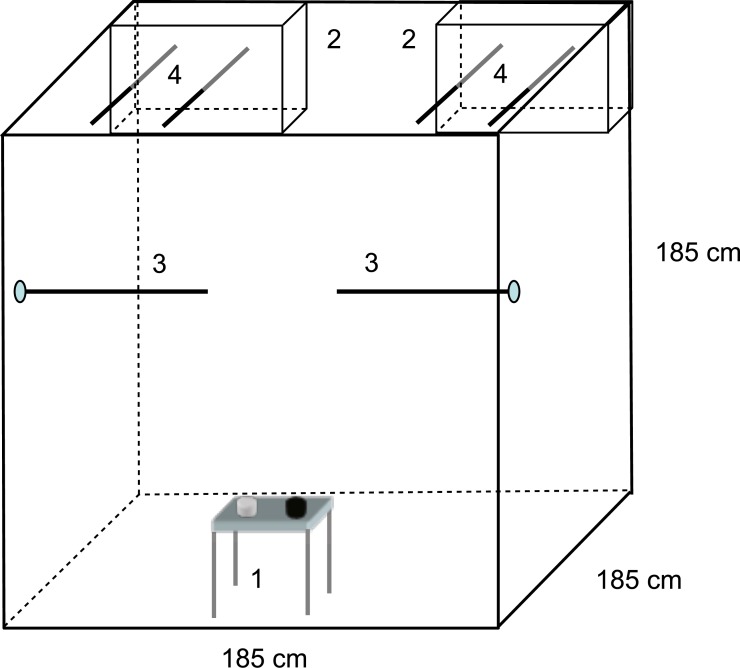
Mate choice experimental setup. Choosers (males or females) could move freely in the large free-flight aviary. Two cages (2) with individuals of the opposite sex than the chooser were placed in the top rear corners of the aviary. Choosers were provided with food and water (1) and perches in a neutral zone (3). Perches of the cages extended into the aviary (4) so that choosers could perch directly in front of one stimulus bird (preference zone) without being able to see the other stimulus bird. Stimulus birds in the cages could not see each other due to a visual barrier between cages. Drawings are only partly to scale. See text for further details. Figure from Naguib & Nemitz (2007), 10.1371/journal.pone.0000901.g004.

### Mate-choice experiment

The mate choice experiments were conducted in a free flight indoor aviary (185 × 185 × 185 cm) in which the choosing bird could move around freely (Fig. 1). Dyads of stimulus birds from the early treatment groups were presented each in a cage (20 × 40 × 30 cm) with wire mesh sides, positioned in the top corners on one side of the aviaries. The sides of the cages facing each other were covered, so that the two stimulus birds could not see each other (Naguib & Nemitz, 2007). Each cage had a perch in front of it, from which the choosing bird could see only the subject in front it but not the other subject. Use of that perch was scored as choice for that stimulus bird.

Choosers were 38 adult females and 44 adult males, which were not exposed to the nutritional treatments and had been raised under the intermediate diet described above throughout and were randomly selected from the Bielefeld zebra finch colony. Stimuli birds were adult offspring originating from the breeding experiment described above (Honarmand, Goymann & Naguib, 2010).

Two weeks prior to the mate choice experiments all subjects were transferred to cages (83 × 30 × 40 cm) situated in the experimental room. Cages contained four to six birds of the same sex. All birds could vocally interact with each other at any time but could not visually interact with individuals outside their cage. After the experiment, females were transferred to an indoor aviary while males stayed in the cages until their song was recorded.

Each of the 82 choosers was tested with a randomly chosen unique stimulus bird-dyad of the opposite sex, raised under the experimental conditions described above. With a total of 82 stimulus bird dyads (44 females, 38 males) all three possible treatment combinations were tested in random order. Assignment of birds as stimulus dyad and as choosers was controlled for genetic relatedness and song tutoring, hence combinations with relatives and same song tutors were prevented. Stimulus birds had 20 min to acclimatize inside the test cages of the choice aviary, then a chooser was placed inside the start box (20 × 20 × 20 cm) and the observer (MH) hid in an observation shed equipped with a small one-way mirror. A 20 min observation period started when the chooser’s start box was opened via a remotely controlled string. Noldus Observer (Basic 5.0) was used to record exact time spent in front of the cages and to sample singing in 10 s intervals. Choosers and subjects had access to *ad libitum* food and water at any time during the experiment (Fig. 1). The mate choice aviary was situated in the same experimental room as described above, but without visual contact between the test birds and the other birds in the same room. Cheek patch size an indicator of plumage development was taken into the analysis as an attractiveness measure. Cheek value was measured at day 65 as described in Honarmand, Goymann & Naguib (2010) which in brief was the ratio of the mean number of pixels per cheek patch and the mean number of pixels per beak (Leader & Nottebohm, 2006; Honarmand, Goymann & Naguib, 2010).

### Fecundity

Subjects were 41 males and 42 females raised in the above described breeding experiment. As part of a subsequent breeding experiment from August 2007 to January 2008 subjects as adults were paired randomly to opposite sex individuals from the colony, and which had not been part of this experiment, controlling for relatedness (at least to great-grandparents). In this breeding experiment we here determined the effects of early developmental treatments on latencies to nest building and egg laying, clutch size, hatching success, and brood size. Breeding was similar to the above-mentioned breeding experiment until the hatching of chicks. Nests were checked daily between 0900 and 1100 h for nest building, egg laying, and hatching. New hatchlings were weighed to the nearest 0.01 g (Sartorius PT120) and individually marked by cutting down the down feathers (Adam, Scharff & Honarmand, 2014). All pairs here received high quality food during this period and from day three post-hatching were exposed to different experimental treatments, which accordingly are not considered here.

### Statistical analysis

In the female choice tests, we tested female preferences in relation to male early developmental diet and male singing during the tests using a linear mixed effect model with the time spent in front of a male as dependent variable and as explanatory variables, the number of songs, the three different experiments (LH-HL, LH-HH, HL-HH), the treatment of a male within each test (lower or higher nutritional quality with LH being considered as lower quality than HL), and their interaction. Male identity nested in stimulus test pair was used as random effect. The same model construction was used for effects of cheek patch size and in the male choice tests.

For the fecundity tests, treatment effects were analyzed separately for the sexes for the latency to egg laying, clutch size hatching success, the number of hatchlings with Kruskal Wallis *X*^2^-tests. Linear mixed effect models for offspring hatching mass were calculated separately for the offspring where either the female or the male was raised in this experiment under the different nutritional conditions, with maternal (or paternal) treatment, maternal (or paternal) fledgling body mass, maternal (or paternal) breeding body mass, father adolescent cheek patch size and offspring sex as fixed effects. Parent ID was used as random effect. Residuals in final models were tested for normal distribution (Shapiro Wilks test) and data were transformed when deviations were significant. Final models were determined using stepwise backward elimination of non-significant terms. All statistics were run in R 3.3.3 for Mac OsX (R Core Team, 2017).

### Ethical note

Housing and breeding of the birds was permitted by the local authorities (Bezirksregierung Detmold, 50.05.03.1.1 (I/04), 04.10.2004). After the experiments all birds remained in the lab stock.

## Results

**Figure 2:**
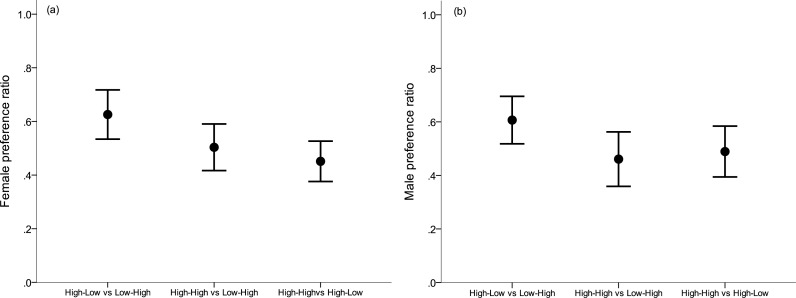
Preference ratios. (A) Female and (B) male preference ratio for males from the better nutritional conditions, respectively (mean ± s.e.). A preference ratio of 0.5 indicates equal amount of time spent at either opposite sex individual. Early nutritional treatment groups in which subjects were raised during their first month post hatching: LH (lower quality food as nestling-higher quality food as fledgling), HL (higher–lower) and HH (higher–higher).

Females showed no significant preference for males that were raised in the better conditions in any of the three mate choice experiments (LME: treatment (lower or higher) *F*_1,36_ = 0.19, *p* = 0.89; Experiment (the three different combinations) *F*_2,35_ = 0.37, *p* = 0.69; Interaction between treatment and experiment; *F*_2,32_ = 0.73, *p* = 0.49; Fig. 2A). Likewise males showed no significant preference for females that were raised in the better conditions in any of the three mate choice experiments (LME: treatment (lower or higher) *F*_1,41_ = 0.01, *p* = 0.93; Experiment (the three different combinations) *F*_2,39_ = 0.85, *p* = 0.44; Interaction between treatment and experiment; *F*_2,39_ = 1.71, *p* = 0.19; Fig. 2B).

**Figure 3:**
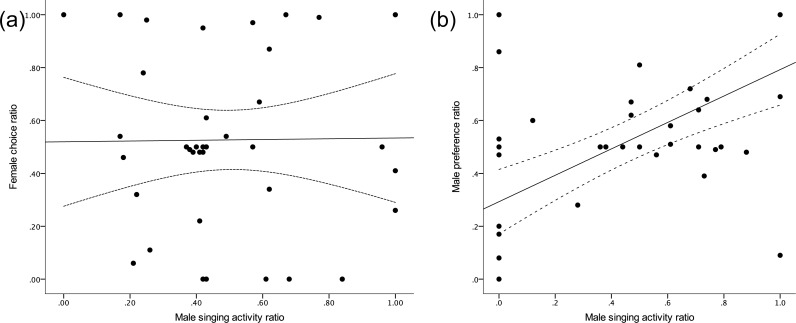
(A) Female preference for males of the higher quality nutritional treatment, respectively, did not correlate with singing proportion of the males in the female choice tests (B). In the male choice tests, i.e., when males could actively choose a female, males sang more in front of their preferred female. Regression lines with confidence intervals (hatched lines) to visualize relations.

In the female choice experiments there was no significant relation between female choice and male singing (LME: *F*_1,37_ = 1.05, *p* = 0.31; Fig. 3A) or male cheek patch size (LME: *F*_1,33_ = 0.44, *p* = 0.51). In the male choice experiments, i.e., when a male could actively choose between females, however, male singing activity was positively associated with presence in front of a female (LME: *F*_1,43_ = 19.21, *p* < 0.001, Fig. 3B).

**Figure 4:**
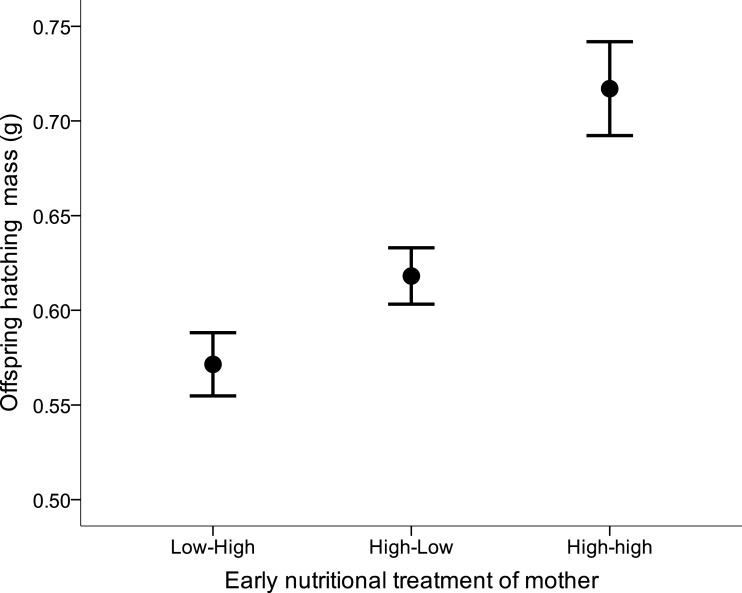
Offspring body mass (mean ± s.e.) from mothers which were raised under different early nutritional conditions as nestling and/or fledglings (LH, lower quality food as nestling-higher quality food as fledgling, HL, higher-lower, HH, higher-higher).

There was no effect of either the female’s or the male’s former nutritional treatment on the latency to egg laying, clutch size, hatching success, the number of hatchlings, or sex ratio at hatching (all Kruskal–Wallis *x*^2^ < 4.99, *df* = 2, all *p* > 0.08). Yet, maternal early nutritional treatments had a strong effect on subsequent offspring’s body mass at hatching (LME: *F*_2,28_ = 8.76, *p* = 0.001). Offspring from LH and HL females were lighter than offspring from HH females (Fig. 4). There also was a trend of female own fledgling mass on her offspring mass at hatching (LME: *F*_1,28_ = 3.83, *p* = 0.060). Neither paternal nutritional treatment nor any of the paternal traits affected offspring mass at hatching (paternal nutritional treatment, *F*_2,18_ = 0.01, *p* = 0.99).

## Discussion

Nutritional treatments during the different periods in early development did not affect male or female attractiveness in adulthood, nor did they affect male singing during these mate choice trials. Moreover, female preference for a male was not associated with male singing activity. Nutritional conditions under which females were raised also did not affect the latency to egg laying or the number of offspring once they were allowed to reproduce themselves. However, hatchlings from females, which had been raised under poorer nutritional conditions either as nestlings or as fledglings, were significantly lighter than offspring from females raised under better nutritional conditions throughout development.

Our findings that neither female nor male attractiveness was influenced by early nutritional treatments contrasts our predictions and indicates that individual attractiveness was not affected by developmental constraints; a result that is consistent with research from other vertebrate species (Walling et al., 2007). Indeed, the evidence that early nutritional conditions affect attractiveness in zebra finches is mixed. De Kogel & Prijs (1996) found in a brood manipulation experiment, in which birds were kept until day 50 in experimental groups, so beyond nutritional independence, that male visual ornaments, song, and attractiveness were affected by the brood size manipulation (De Kogel & Prijs, 1996). Naguib, Heim & Gil (2008), using brood size manipulation until day 35, i.e., until nutritional independence, did not find such effects. Also Blount and colleagues (2003), who experimentally manipulated nutritional quality during a 15 day period post hatching, did not find effects on neither biometry nor male attractiveness. Woodgate and colleagues (2010) found differences in activity in a mate choice task related to early nutritional treatments but also not in the resulting preference (Woodgate et al., 2010). Holveck, Geberzahn & Riebel (2011) showed that females prefer males from similar developmental background while males did not shown such preferences. Furthermore, males that experienced poor nutritional conditions during the first month of life compensate initial reduced cheek patch sizes until sexual maturation (Krause & Naguib, 2015). Yet when nutritional conditions were manipulated during adolescence, the period when song is learnt and plumage ornaments develop, birds from better conditions showed better plumage ornaments development and were more attractive in mate choice tests (Naguib & Nemitz, 2007). Thus, there is increasing evidence that nutritional stress experienced prior to development of sexual ornaments does not necessarily have negative effects on attractiveness in such laboratory choice conditions. Yet a range of studies showed that early developmental conditions do affect the expression of song traits (Spencer et al., 2003; Buchanan et al., 2004; Spencer et al., 2005) and female preference for song traits (Riebel, 2009; Riebel, Naguib & Gil, 2009; Holveck & Riebel, 2010) even though not all studies found early developmental effects on song complexity or song preferences (Gil et al., 2006; Brumm, Zollinger & Slater, 2009; Honarmand, Riebel & Naguib, 2015). We did not quantify the effects of the early nutritional treatments applied here on male song traits and on song preferences, yet it is likely that traits other than song also will be involved in mate choice decisions.

Unlike most previous studies, this study included attractiveness tests for both sexes, showing that also choosing males did not discriminate between females from different nutritional backgrounds. Especially in a species with biparental brood care as the zebra finch, mutual mate choice is expected even though male choice may be cryptic (Engqvist & Sauer, 2001; Kokko & Johnstone, 2002). Thus, females would equally benefit, as do males, by not losing in attractiveness for limitations experienced during development. Moreover, zebra finches are very faithful (Griffith et al., 2010) and both parents invest similarly in raising the offspring (Gilby et al., 2011; Mariette & Griffith, 2012; Gilby, Mainwaring & Griffith, 2013; Morvai et al., 2016) so that indeed both partners should be choosy. Even though males sang more in front of their preferred female, females did not show a significant preference for males with a high singing activity. Singing activity has not been a good predictor for female preference also in other studies (Riebel, 2009) and indeed may function beyond being a signal in mate choice, given that males in the wild continue singing substantially towards their mate after mating (Dunn & Zann, 1996; M Naguib & S Griffith, pers. obs., 2016).

The dietary effects during early development had profound but only transient effects on biometry and physiology (Honarmand, Goymann & Naguib, 2010). As adults there was no remaining effect of early conditions on biometry (Honarmand, Goymann & Naguib, 2010) and likewise here we did not find effects on the latency to breed when given the opportunity later on in life. Also, in a previous brood size manipulation experiment, no such effects of early conditions experienced on subsequent egg laying were found(Gil et al., 2004). This contrasts with Blount and colleagues (2006), who showed delayed egg laying when assortatively mating males and females were raised in the first two weeks post hatching under poorer nutritional conditions. Breeding pairs in our experiment were not mated assortatively, which might in part explain the difference with the experiment by Blount et al. (2006). To what extend findings on mate preferences in captivity, as conducted here, reveal decision processes under the more complex natural conditions remains an important topic to be addressed by future studies. In the wild, pairs are expected to make a joint decision when to breed and subsequent behavioural synchrony affects reproductive success (Mariette & Griffith, 2012; Mariette & Griffith, 2015). Possibly it is then more important to become compatible with a partner over time rather than choosing a partner with specific traits or developmental background. Indeed, the timing of laying eggs can be important for improving individual fitness and breeding pairs are expected to delay laying only when environmental conditions and their physiological and reproductive state suggest to do so (Blount et al., 2006). Zebra finches are opportunistic breeders and under natural conditions benefit by breeding as soon as conditions are appropriate as their fitness will strongly be affected by the success of subsequent breeding events as long as conditions stay acceptable (Immelmann, 1965; Zann, 1996).

Our findings of the second breeding experiment that the early conditions experienced affected the body mass of subsequent offspring, provide further evidence in zebra finches for trans-generational effects of conditions experienced during early development (Naguib & Gil, 2005; Naguib, Nemitz & Gil, 2006; Krause & Naguib, 2014). Females, which were raised on a low quality diet as nestlings or fledglings later on in life, produced lighter hatchlings compared to females, which experienced higher quality food throughout their first month post hatching. As egg mass predicts hatchling mass (Krause, Krüger & Schielzeth, 2017), it is possible that females raised under poorer early life conditions may have laid smaller eggs which then may have led to the observed lower hatching mass of their offspring. Likewise, females may have affected offspring development by investing less testosterone in eggs (Gil et al., 2004). This possibility would explain why no such hatching mass effects were found for the offspring when their father had been raised under poorer nutritional conditions. Given that birds in the second breeding experiment bred under good nutritional conditions, it may well be that the trans-generational effect in part was due to a matched environment as only the birds from the high quality food treatment bred under equal conditions they were raised in, while birds from the lower quality conditions bred under conditions not matching their own developmental period. Such effects of matched environments have been shown also in other species (Taborsky, 2017) and have been discussed as an adaptive programming along the thrifty phenotype hypothesis to perform best in the environment experienced in early life (Wells, 2007). Yet, in an experiment specifically designed to test these ideas in zebra finches we did not find that birds in matching environment had fitness benefits (Krause & Naguib, 2014; Krause, Krüger & Schielzeth, 2017). Indeed, as zebra finches breed opportunistically in unpredictable environments, such programming might not be as adaptive as it is for animals living in more predictable environments.

In conclusion, short periods of food limitations are likely to occur in the wild and it appears to be a good strategy to compensate for any potential phenotypic deficits as much as possible. Costs of compensation are likely reappear only in contexts that are challenging to individuals.

##  Supplemental Information

10.7717/peerj.3628/supp-1 Supplemental Information 1 Data S1

## Additional Information and Declarations

### Data Availability

The following information was supplied regarding data availability: The raw data has been uploaded as a Supplementary File.
